# Diagnostic value of pentraxin 3 in respiratory tract infections: A meta-analysis

**DOI:** 10.1097/MD.0000000000019532

**Published:** 2020-04-03

**Authors:** Wu Ye, Qing-Dong Huang, Ting-Yu Tang, Guang-Yue Qin

**Affiliations:** Department of Respiratory Diseases, Zhejiang Hospital, Hangzhou, Zhejiang Province, People's Republic of China.

**Keywords:** diagnostic test, meta-analysis, pentraxin 3, respiratory tract infections, ventilator-associated pneumonia

## Abstract

**Background:**

Pentraxin 3 is an acute inflammatory protein of the long pentraxin subfamily. A meta-analysis was performed to assess diagnostic accuracy of pentraxin 3 for respiratory tract infections.

**Methods:**

We identify studies examining diagnostic value of pentraxin 3 for respiratory tract infections by searching Pubmed, Web of Knowledge, and Cochrane Library. The sensitivity, specificity, negative likelihood ratio (LR), positive LR, and diagnostic odds ratio were pooled. The area under the summary receiver operator characteristic (SROC) curve and Q point value (Q∗) were calculated.

**Results:**

A total of 8 studies with 961 individuals were eligible for this meta-analysis. The pooled sensitivity of pentraxin 3 in diagnosis of respiratory tract infections was 0.78, the pooled specificity was 0.73, the area under the SROC curve was 0.84, and the Q∗ was 0.77. The area under the SROC curve of serum and bronchoalveolar lavage fluid (BALF) pentraxin 3 was 0.85 and 0.89, respectively. Meta-regression analysis revealed that cutoff value was the source of heterogeneity among the included studies. The Deek funnel plot test suggested no evidence of publication bias. Subgroup analyses showed that the area under the SROC curve of pentraxin 3 in diagnosis of ventilator-associated pneumonia (VAP) was 0.89.

**Conclusion:**

Pentraxin 3 has a moderate accuracy for diagnosing respiratory tract infections and VAP. The overall diagnostic value of BALF level of pentraxin 3 is superior to its serum concentration.

## 1 Introduction

Respiratory tract infections are major causes of morbidity, hospitalization, and mortality worldwide, particularly in elderly and children.^[1,2]^ Early diagnosis of respiratory tract infections and assessment of disease severity are essential for optimal treatment. The use of traditional microbial culture has limitations, including inadequate sensitivity and difficulty in identifying colonization.^[3]^ The absence of reliable tools for diagnosing respiratory tract infections remains a major challenge. Many biomarkers have been developed to improve the diagnostic accuracy such as C-reactive protein, procalcitonin, erythrocyte sedimentation rate (ESR), soluble triggering receptor expressed on myeloid cells-1, soluble urokinase-type plasminogen activator receptor, plasminogen activation inhibitor-1, and pentraxin 3.^[3,4]^

Pentraxin 3 is an acute inflammatory protein of the long pentraxin subfamily.^[5]^ The classic short pentraxin C-reactive protein is produced in the liver and induced by proinflammatory cytokines such as interleukin 6.^[5]^ Unlike C-reactive protein, pentraxin 3 can be rapidly produced by neutrophils, mononuclear phagocytes, and myeloid dendritic cells in response to pathogens.^[4,6]^ Pentraxin 3 upregulates the tissue factor in monocytes and promotes the recruitment of neutrophils.^[4,7]^ Circulating pentraxin 3 concentrations are low in normal conditions, and rapidly increase during inflammation.^[8]^ Pentraxin 3 is elevated earlier than C-reactive protein in acute lung injury. Increased pentraxin 3 levels are correlated with disease severity and mortality in patients with acute lung injury and sepsis.^[9]^

Recent studies show that pentraxin 3 has emerged as a promising marker for diagnosing respiratory tract infections.^[9]^ Therefore, we performed a meta-analysis of eligible clinical studies to assess diagnostic value of pentraxin 3 in respiratory tract infections and ventilator-associated pneumonia (VAP).

## 2 Materials and methods

### 2.1 Search strategy

We searched PubMed, Web of Knowledge, and Cochrane Library to identify studies examining diagnostic accuracy of pentraxin 3 for respiratory tract infections published up to August 2019. References of retrieved studies and relevant reviews were manually examined. The following keywords were used: “pentraxin 3,” “PTX3 protein,” “respiratory tract infections,” “pneumonia,” “respiratory infections,” and “pulmonary infections.” Ethical approval was not required, as all analyses were based on previous published studies.

### 2.2 Study selection

Studies were included if they fulfilled the following criteria: (1) original articles published in English; (2) studies limited to human subjects; (3) papers assessing diagnostic accuracy of pentraxin 3 for respiratory tract infections; (4) studies provided sufficient data to construct the 2 × 2 contingency table, and calculate sensitivity and specificity. Studies with fewer than 10 individuals were excluded.

### 2.3 Data extraction and quality assessment

Two authors (Wu Ye and Tingyu Tang) independently reviewed the included studies and obtained relevant information. If disagreement occurred, 2 authors reexamined discrepancies and resolved by consensus. The following data were extracted: family name of the first author; publication year; region of the study performed; age and number of study population; prevalence and category of respiratory tract infections; sample source; assay method for pentraxin 3; cutoff level; and number of true positive (TP), false positive (FP), false negative (FN), and true negative (TN).

Two reviewers (Wu Ye and Tingyu Tang) independently assessed the methodological quality of included trials using the Quality Assessment of Diagnostic Accuracy Studies (QUADAS).^[10]^ We attributed a score of 1 point for each “yes,” 0.5 point for each “unclear,” and 0 point for each “no.” The maximum score is 14 point.

### 2.4 Statistical analysis

All statistical analyses were performed using the MetaDisc version 1.4 software (Clinical Biostatistics Team, Ramón y Cajal Hospital, Madrid, Spain) and Stata version 16.0 software (StataCorp, College Station, TX). The sensitivity, specificity, negative likelihood ratio (LR), positive LR, and diagnostic odds ratio (DOR) were pooled, the summary receiver operator characteristic (SROC) curves were constructed, and the area under the SROC curve and Q point value (Q∗) were calculated. The heterogeneity among included studies was evaluated by the Chi-square test. If heterogeneity was present, meta-regression analyses were performed to explore the sources of heterogeneity. The presence of publication bias was tested using the Deek funnel plot. A *P* value < .05 was considered statistically significant.

## 3 Results

### 3.1 Study characteristics

**Figure 1:**
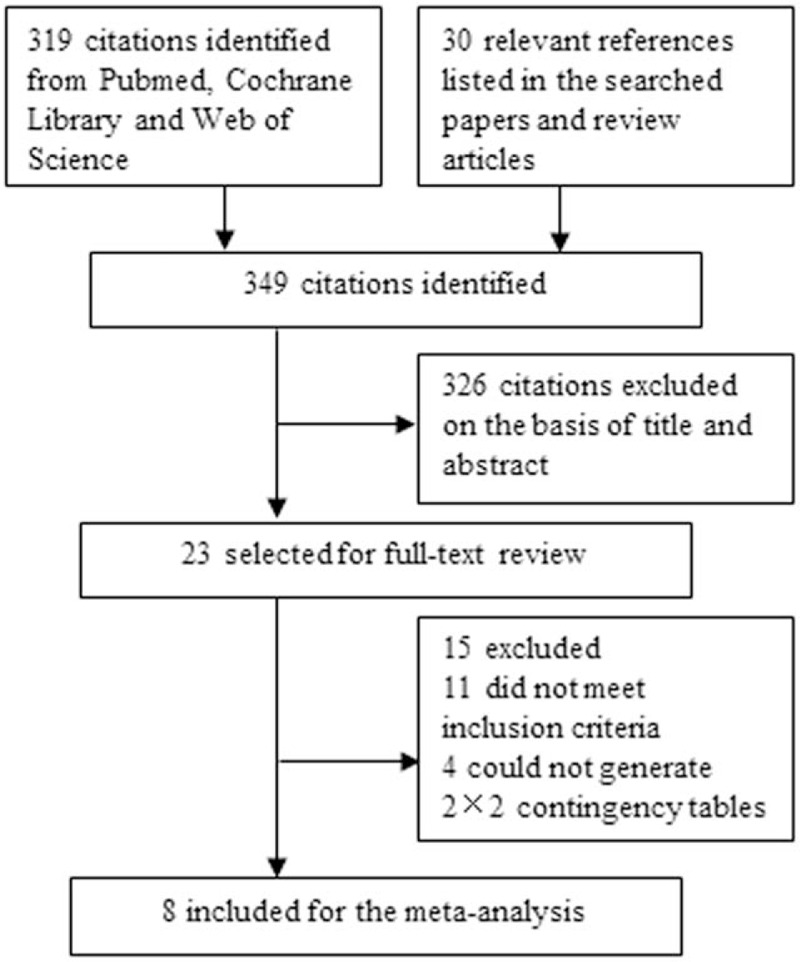
Flowchart of the study selection process.

Our initial literature search yielded 349 studies (Fig. 1). Three hundred twenty-six citations were excluded after review of title and abstract. Of 23 studies selected for full-text assessment, 11 did not meet inclusion criteria, and 4 could not generate the 2 × 2 contingency table. Ultimately, a total of 8 studies with 961 individuals were eligible for this meta-analysis.^[9,11–17]^

The clinical characteristics of included studies are presented in Table 1. Serum specimens were collected in 5 studies, sputum was for 1 trial and bronchoalveolar lavage fluid (BALF) was gathered in the other 4 studies. Two studies tested both BALF and serum samples,^[14,17]^ 3 trials only collected serum, 2 studies only detected BALF, and the other study obtained sputum. The concentration of pentraxin 3 was measured by the enzyme-linked immunosorbent assay (ELISA) in 7 studies, while immunostaining was performed in only 1 study. Commercial ELISA kits were used to measure pentraxin 3 levels according to the manufacturer's instructions. The cutoff levels of pentraxin 3 among the included studies were ranged from 0.312 ng/mL to 118 ng/mL. In our meta-analysis, the QUADAS scores for included studies were all above 10, indicating that all studies were of high quality.

**Table 1:**
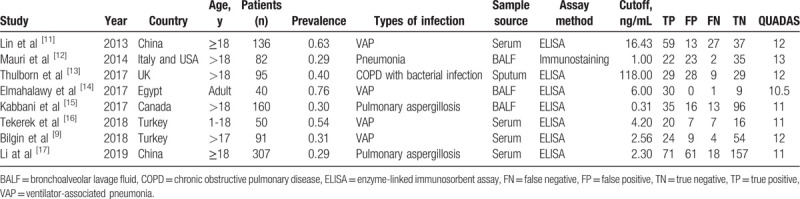
Clinical characteristics of included studies.

### 3.2 Diagnostic accuracy for respiratory tract infections

**Figure 2:**
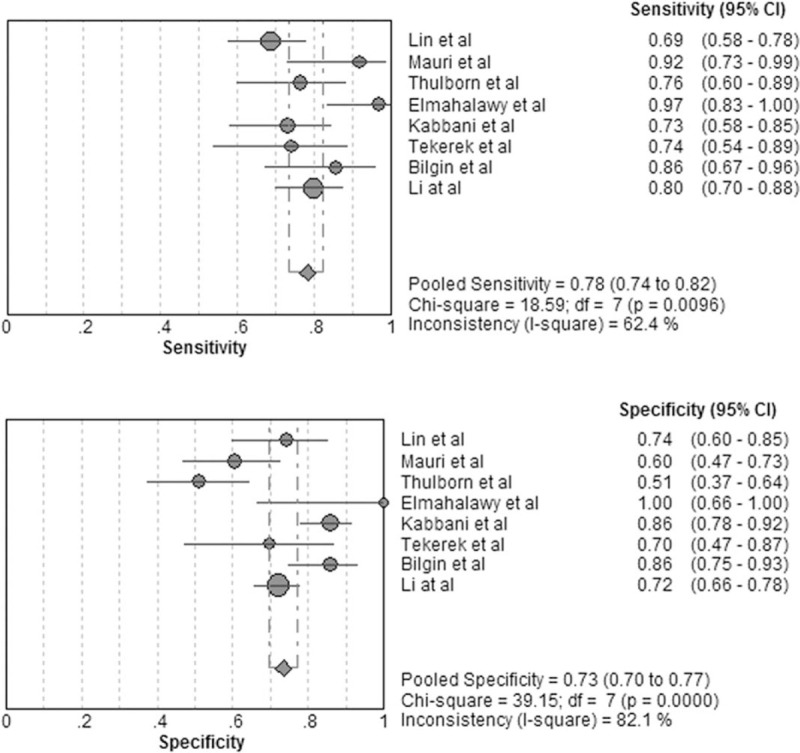
Forest plot of sensitivity and specificity for pentraxin 3 in diagnosis of respiratory tract infections. The pooled sensitivity was 0.78 (95% CI, 0.74–0.82) and the pooled specificity was 0.73 (95% CI, 0.70–0.77).

**Figure 3:**
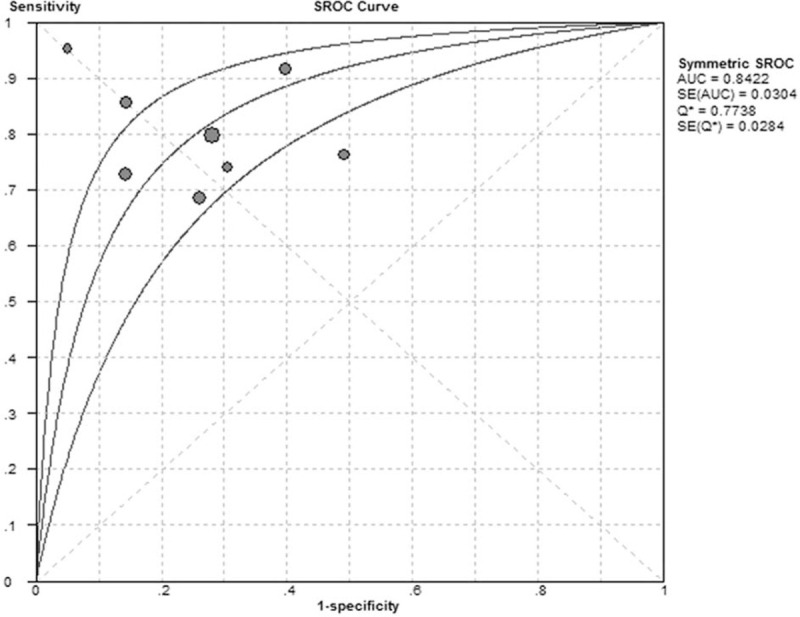
Summary receiver operating characteristic (SROC) curve of pentraxin 3 in diagnosis of respiratory tract infections. The area under the SROC curve was 0.84 and the Q^∗^ was 0.77.

The forest plot for sensitivity and specificity of pentraxin 3 in diagnosis of respiratory tract infections is presented in Fig. 2. The pooled sensitivity was 0.78 [95% confidence interval (CI), 0.74–0.82] and the pooled specificity was 0.73 (95% CI, 0.70–0.77). The positive LR was 2.94 (95% CI, 2.11–2.10), the negative LR was 0.30 (95% CI, 0.22–0.41), and the DOR was 10.84 (95% CI, 6.02–19.51). As shown in Fig. 3, the area under the SROC curve was 0.84 and the Q∗ was 0.77, indicating a moderate diagnostic accuracy.

### 3.3 Heterogeneity assessment and meta-regression analysis

The *I*^2^ for pooled sensitivity, specificity, and DOR was 62.4%, 82.1%, and 60.7%, respectively. The results indicated substantial heterogeneity among the included studies. We performed meta-regression analysis to explore the sources of potential heterogeneity. The relevant covariates included age (≥18 years vs <18 years), sample source (serum, sputum vs BALF), assay method (immunostaining vs ELISA), cutoff value (≥10 ng/mL vs <10 ng/mL). The meta-regression analysis showed that the source of heterogeneity among studies was related to the cutoff value (*P* = .04).

### 3.4 Publication bias

**Figure 4:**
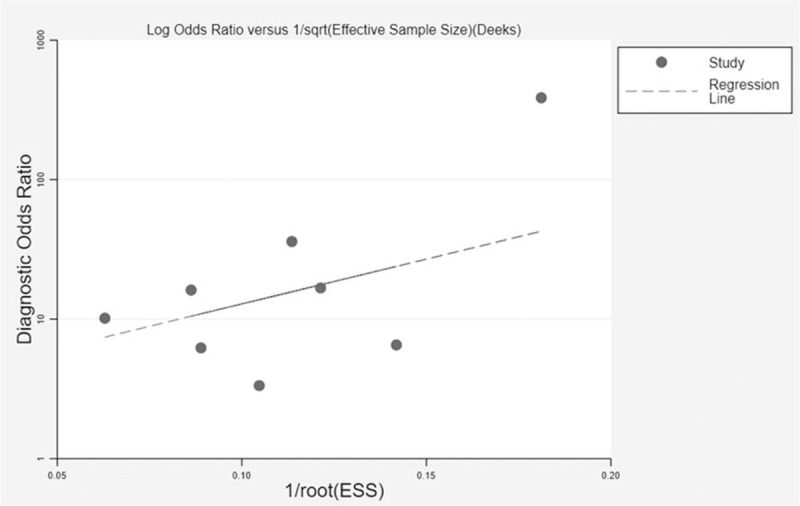
The Deek funnel plot for assessment of publication bias. No publication bias was found among the included studies.

The Deek funnel plot test suggested no evidence of publication bias among the included studies (*P* = .25, Fig. 4).

### 3.5 Subgroup analysis

**Figure 5:**
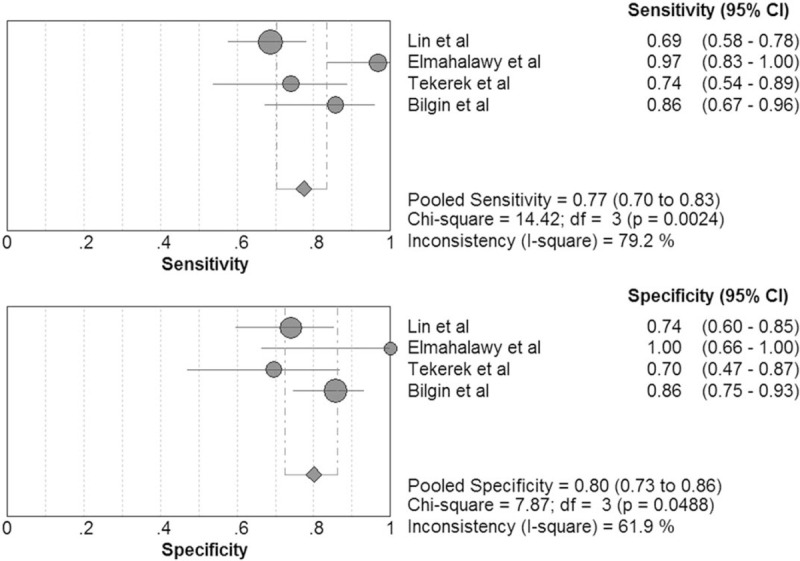
Forest plot of sensitivity and specificity for pentraxin 3 in diagnosis of ventilator-associated pneumonia (VAP). The pooled sensitivity was 0.77 (95% CI, 0.70–0.83) and the pooled specificity was 0.80 (95% CI, 0.73–0.86).

The pooled sensitivity of pentraxin 3 in VAP diagnosis was 0.77 (95% CI, 0.70–0.83) and the pooled specificity was 0.80 (95% CI, 0.73–0.86; Fig. 5). The positive LR was 3.59 (95% CI, 2.04–6.34), the negative LR was 0.24 (95% CI, 0.12–0.51), the DOR was 15.92 (95% CI, 4.51–56.15), the area under the SROC curve was 0.89, and the Q∗ was 0.82.

**Figure 6:**
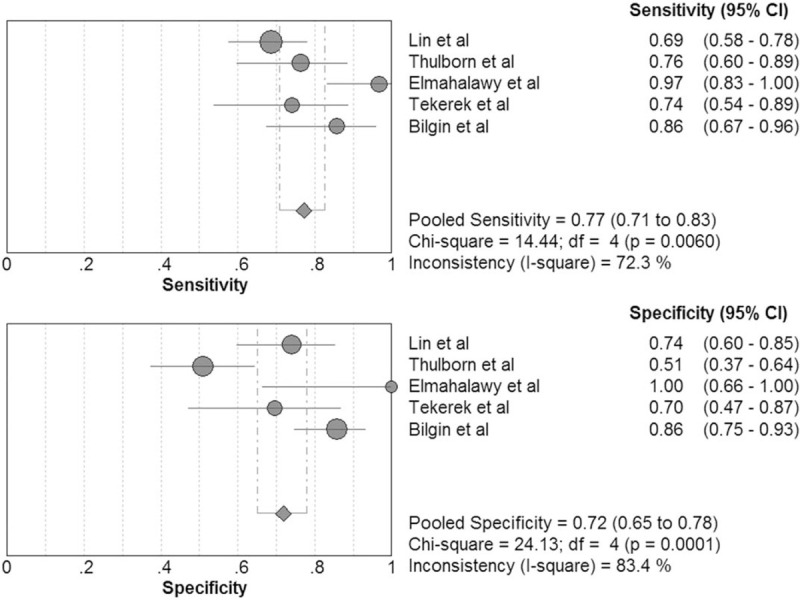
Forest plot of sensitivity and specificity for pentraxin 3 in diagnosis of bacterial respiratory tract infections. The pooled sensitivity was 0.77 (95% CI, 0.71–0.83) and the pooled specificity was 0.72 (95% CI, 0.65–0.78).

The pooled sensitivity of pentraxin 3 in diagnosis of bacterial respiratory tract infections was 0.77 (95% CI, 0.71–0.83) and the pooled specificity was 0.72 (95% CI, 0.65–0.78; Fig. 6). The positive LR, negative LR, DOR, area under the SROC curve, and Q∗ was 2.95 (95% CI, 1.59–5.47), 0.30 (95% CI, 0.18–0.51), 10.60 (95% CI, 3.76–29.87), 0.89, and 0.80, respectively.

**Figure 7:**
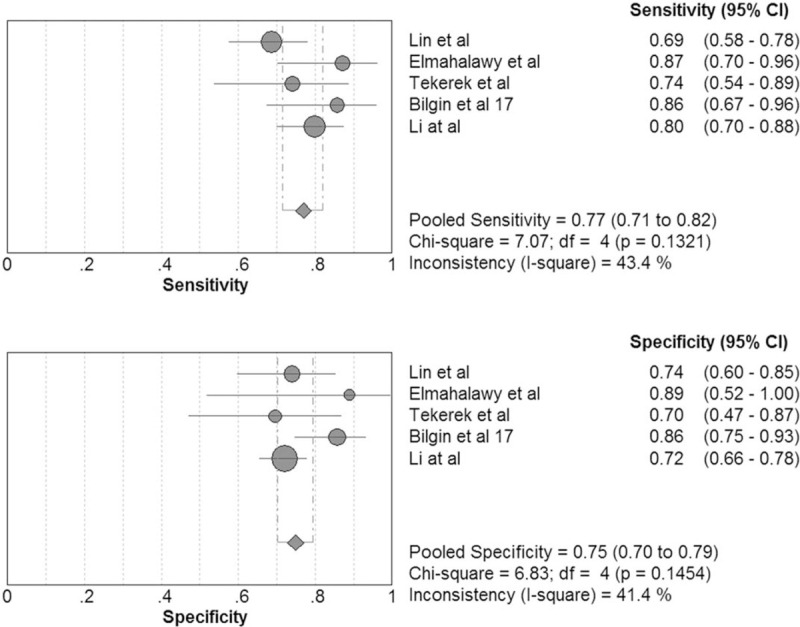
Forest plot of sensitivity and specificity for serum pentraxin 3 in diagnosis of respiratory tract infections. The pooled sensitivity was 0.77 (95% CI, 0.71–0.82) and the pooled specificity 0.75 (95% CI, 0.70–0.79).

The pooled sensitivity and specificity of serum pentraxin 3 for diagnosing respiratory tract infections was 0.77 (95% CI, 0.71–0.82) and 0.75 (95% CI, 0.70–0.79; Fig. 7), respectively. The positive LR was 3.19 (95% CI, 2.33–4.38), the negative LR was 0.29 (95% CI, 0.20–0.43), and the DOR was 11.35 (95% CI, 5.91–21.78). The area under the SROC curve was 0.85 and the Q∗ was 0.78.

**Figure 8:**
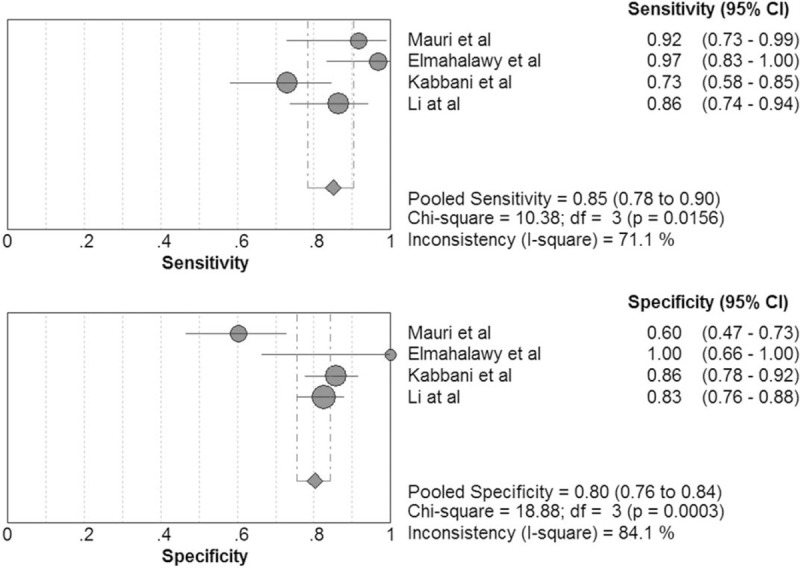
Forest plot of sensitivity and specificity for BALF pentraxin 3 in diagnosis of respiratory tract infections. The pooled sensitivity was 0.85 (95% CI, 0.78–0.90) and the pooled specificity was 0.80 (95% CI, 0.76–0.84).

The pooled sensitivity of BALF pentraxin 3 for diagnosis of respiratory tract infections was 0.85 (95% CI, 0.78–0.90) and the pooled specificity was 0.80 (95% CI, 0.76–0.84; Fig. 8). The positive LR, negative LR, and DOR was 4.09 (95% CI, 2.29–7.31), 0.18 (95% CI, 0.09–0.35), and 23.41 (95% CI, 11.64–47.09), respectively. The area under the SROC curve was 0.89 and the Q∗ was 0.83.

## 4 Discussion

Respiratory tract infections remain the most common reason for patients to seek medical service.^[18,19]^ A rapid and accurate approach to diagnose respiratory tract infections is crucial for starting appropriate treatment.^[18,19]^ Pentraxin 3, the first identified member of the long pentraxin subfamily, is a vital component of innate immunity related to sepsis.^[4,6,20]^ Pentraxin 3 plays an important role in the early stages of inflammation by recognizing microorganisms, promoting pathogen recognition and regulating complement activation.^[5,21,22]^ To our knowledge, no meta-analysis to date has assessed diagnostic value of pentraxin 3 in respiratory tract infections. In the present study, we included 8 eligible trials with 961 patients to estimate the test value of pentraxin 3 for respiratory tract infections and performed subgroup analyses to explore diagnostic accuracy for VAP and bacterial respiratory tract infections.

Our meta-analysis shows that the pooled sensitivity of pentraxin 3 in diagnosis of respiratory tract infections was 0.78(CI, 0.74–0.82) and the pooled specificity was 0.73 (95% CI, 0.70–0.77). In forest plot, most included studies agreed on the pooled sensitivity and specificity except 2 studies.^[13,14]^ The reported sensitivity and specificity in the study by Elmahalawy et al^[14]^ was 96.8% and 100%, respectively. The results were based on a small population with 40 patients. In the study by Thulborn et al,^[13]^ the diagnostic specificity of pentraxin 3 was 50.9%. Of the 8 included studies, only this study^[13]^ measured pentraxin 3 concentrations in sputum. In the present meta-analysis, QUADAS scores of included studies are all above 10, which suggested that the qualities of all trials are high.

The SROC curve illustrates a summary of overall test accuracy. The area under the SROC curve ranging 0.50 to 0.70 represented low accuracy, 0.70 to 0.90 suggested moderate accuracy, and >0.90 revealed high accuracy.^[23,24]^ In the present study, the area under the SROC curve was 0.84 and the Q∗ was 0.77, indicating that pentraxin 3 had a moderate accuracy for diagnosing respiratory tract infections. Our subgroup analyses suggested that overall diagnostic accuracy of pentraxin 3 was similar for VAP and bacterial respiratory tract infections.

BALF is a common source of samples for diagnosing lung infections. The main advantage of BALF is that it is close to the site of lung infections and can be a good indicator of the local lung environment.^[25,26]^ BALF and serum biomarkers may provide different diagnostic values. In the present meta-analysis, the pooled sensitivity and specificity of serum pentraxin 3 in diagnosis of respiratory tract infections was 0.77 and 0.75, respectively. The pooled sensitivity of BALF pentraxin 3 was 0.85 and the specificity was 0.80. Among the included studies, 2 studies^[14,17]^ examined both BALF and serum samples, and both found that BALF pentraxin 3 has a diagnostic value superior to serum pentraxin 3. These results indicated that the overall diagnostic accuracy of pentraxin 3 in BALF was better than that in serum. BALF biomarkers may more accurately reflect lung inflammation.

Heterogeneity among the included studies was evaluated in the current study. The *I*^2^ for pooled sensitivity, specificity, and DOR are all >50%, indicating substantial heterogeneity among included trails. The meta-regression analysis showed that cutoff value was the source of heterogeneity. The cutoff levels of pentraxin 3 were ranged from 0.312 ng/mL to 118 ng/mL in the present meta-analysis. Two studies used cut-off values above 10 ng/mL.^[11,13]^ Samples were collected at different time points in the included studies, which is also a possible source of heterogeneity. Diagnostic studies may have publication bias. Studies with negative results are not easily published, whereas those with positive data are more likely to be published. In the present study, the Deek funnel plot test showed no potential publication bias.

The present study has some limitations. First, pentraxin 3 was calculated by different methods, and its cutoff value varied across included studies, which made it difficult to determine the real diagnostic value. Our meta-regression analysis suggested that cutoff value contributed to heterogeneity. Second, included studies were limited to those in English, which may cause publication bias. Third, respiratory tract infections are made up of many different diseases, which may result in heterogeneity.

In summary, the available evidence suggests that pentraxin 3 has a moderate accuracy for diagnosing respiratory tract infections, VAP, and bacterial respiratory tract infections. BALF level of pentraxin 3 is superior to its serum concentration in diagnosis of respiratory tract infections.

## Author contributions

**Conceptualization:** Wu Ye, Qing-Dong Huang.

**Data curation:** Wu Ye, Ting-Yu Tang.

**Formal analysis:** Wu Ye, Ting-Yu Tang.

**Methodology:** Wu Ye, Ting-Yu Tang.

**Software:** Wu Ye, Ting-Yu Tang.

**Supervision:** Guang-Yue Qin.

**Validation:** Ting-Yu Tang, Guang-Yue Qin.

**Writing – original draft:** Wu Ye, Qing-Dong Huang.

**Writing – review & editing:** Ting-Yu Tang, Guang-Yue Qin.
